# Group B Streptococcus growth in human urine is associated with asymptomatic bacteriuria rather than urinary tract infection and is unaffected by iron sequestration

**DOI:** 10.1099/mic.0.001533

**Published:** 2025-02-20

**Authors:** Deepak S. Ipe, Kelvin G.K. Goh, Devika Desai, Nouri Ben-Zakour, Matthew J. Sullivan, Scott A. Beatson, Glen C. Ulett

**Affiliations:** 1 School of Pharmacy and Medical Sciences, Institute for Biomedicine and Glycomics, Griffith University, Gold Coast Campus, Gold Coast, QLD 4222, Australia; 2 School of Chemistry and Molecular Biosciences, University of Queensland, Brisbane, QLD 4072, Australia; 3 School of Biological Sciences, University of East Anglia, Norwich, NR4 7TJ, UK

**Keywords:** asymptomatic bacteriuria, bacteriuria, *Streptococcus*, urinary tract infection, urine

## Abstract

Group B *Streptococcus* (GBS) causes various infections in adults, including urinary tract infection (UTI) and asymptomatic bacteriuria (ABU). Some bacteria that cause ABU can utilize urine as a substrate for growth, which can promote asymptomatic colonization in the host. An analysis of diverse GBS isolates associated with ABU and UTI for growth in human urine has not been undertaken. Here, we examined a large collection of clinical urinary GBS isolates from individuals with acute UTI (*n*=62), and ABU with bacteriuria ≥10^4^ c.f.u. ml^−1^ (*n*=206) or <10^4^ c.f.u. ml^−1^ (*n*=90) for their ability to grow in human urine. Among all 358 GBS isolates analysed, 40 exhibited robust growth in urine in contrast to 25 that were unable to grow and non-culturable after incubation in urine. Growth phenotypes were disproportionately represented among the different groups of isolates, whereby robust growth was significantly more likely to be associated with high-grade ABU versus low-grade ABU or acute UTI (38/40 vs. 11/25; odds ratio 4.6, 95% CI, 1.5–14.8). Growth of bacteria in urine can depend on iron bioavailability, and we therefore performed growth assays using urine supplemented with 2,2-dipyridyl to chelate iron. In contrast to a control strain of ABU *Escherichia coli,* for which iron limitation significantly attenuated growth, iron sequestration had no significant attenuation effect on the growth of ABU GBS strain 834 in urine. Despite this finding, PCR confirmed the presence of several known growth-associated genes in GBS 834, including *fhuD* for iron uptake. We conclude that GBS adaptation for growth in human urine is more likely to be associated with high-grade ABU than acute UTI, and for GBS 834, this growth trait is not significantly constrained by conditions of iron sequestration.

## Introduction

*Streptococcus agalactiae*, also known as group B *Streptococcus* (GBS), is a Gram-positive commensal bacterium, colonizing the gastrointestinal and genitourinary tracts of up to 50% of healthy adults [1]. GBS is also the leading cause of severe, invasive infection in newborns, pregnant women and older persons with chronic medical illness [2,3]. As an opportunistic pathogen, GBS expresses many virulence factors, including capsular polysaccharide, β-haemolysin/cytolysin, adhesins and stress response factors that mediate subversion of host defence mechanisms [4].

Among the various types of infections that GBS causes in adults are bacteraemia, pneumonia, meningitis and skin and soft tissue infections, including urinary tract infection (UTI) [2,3]. Acute infection caused by GBS can be severe and life-threatening with the overall mortality rate in adults estimated at 15% or more in the USA [3,5, 6]. For UTI, the spectrum of disease includes cystitis, pyelonephritis, urethritis and urosepsis [2,7, 8]. In one study, *S. agalactiae* was cultured from 39% of all cases of symptomatic UTI among nursing home residents >70 years of age [9]. However, GBS also asymptomatically colonizes the female urogenital tract as a commensal [10]. In the urinary tract, GBS can cause asymptomatic bacteriuria (ABU) [11]. It has been suggested that as a pathobiont, GBS may switch from an asymptomatic carriage state to cause pathogenesis as a result of adaptations through genetic mutations (e.g. in certain genes for two-component systems such as *covRS*), or lateral DNA exchange leading to reduced expression of some virulence factors (e.g. capsule) [10].

Bacterial growth in urine is a lifestyle trait for some microbes that colonize the urinary tract, such as *Escherichia coli* and some Gram-positive cocci, including staphylococci and enterococci [12,16]. Bacterial survival in and utilization of urine as a growth substrate is considered an important adaptation strategy for organisms that cause ABU and is thought to promote persistence in the urinary tract [14,16,18]. The bioavailability of nutritional resources in urine, a typically antimicrobial niche, relates to sugars, proteins, amino acids and co-factors such as iron, all of which are critical for bacterial growth [12,15]. Moreover, nutritional immunity acts to limit the bioavailability of such substrates in the host, although highly adapted bacteria subvert this defence strategy by using various mechanisms to acquire nutrients or withstand conditions of stress. For example, low available iron in the host drives bacteria to make siderophores to acquire iron [19,20] and this trait can support survival and/or growth in the urinary tract [21].

The growth of GBS in human urine has been reported for a few clinical isolates, namely ABU 834, 729 and 1014 [22]. However, an analysis of a large, diverse collection of GBS isolates associated with ABU and UTI for fitness for growth in human urine has not previously been undertaken. Here, we examined 358 GBS isolates associated with acute UTI or ABU and compared these for their ability to utilize fresh human urine as a growth medium. In identifying the robust growth of some ABU GBS via consumption of urine, we also tested whether this growth is affected by iron-limiting conditions.

## Methods

### Bacterial isolates

Three hundred and fifty-eight GBS isolates were used in this study; these were originally collected from the urine of patients who presented with UTI or as part of routine screening [8,11]. The isolates were divided into groups of acute UTI (*n*=62), ABU with high-grade (>10^4^ c.f.u. ml^−1^; *n*=206) and ABU with low-grade bacteriuria (<10^4^ c.f.u. ml^−1^; *n*=90) [8,11].

### GBS growth assays in human urine

For each assay, human urine was collected from six healthy volunteers (female and male adults, 1 : 1 ratio) who had not undergone any antibiotic treatment for several weeks prior to urine collection. Equal volumes of urine were pooled, filter sterilized using Millipore filter paper (Millipore corporation, Bedford, CA, USA) with a pore size of 0.45 µm and stored at 4 °C until use. For each experiment, urine was used within 48 h of collection or discarded. For growth assays, the isolates were streaked on Tryptone Soya Agar (TSA; Oxoid, Hampshire, UK) supplemented with 5% horse blood (Serum Australis, NSW) and incubated at 37 °C overnight. Broth cultures of GBS isolates were prepared in Todd–Hewitt broth (THB) with shaking (200 r.p.m.) at 37 °C overnight. Fresh overnight broth cultures were back-diluted (1 : 1000) in PBS pH 7.2, and 2 µl were used to inoculate filter-sterilized, pooled human urine (200 µl) in flat-bottom 96-well plates (Cat. No. TSTPP92096I, SARSTEDT, Ingle Farm, Australia). The growth of isolates was also examined in THB to determine whether isolates were defective for general growth as opposed to the nutrient limited environment of human urine. For measurement of growth in THB, bacterial cells were seeded at the same cell number as in urine growth assays. Duplicate cultures were grown at 37 °C, shaking (200 r.p.m.). OD at 600 nm (OD_600 nm_) was recorded between 0 and 72 h using a POLARstar Omega BMG Labtech plate reader. Duplicate wells containing sterile urine or THB were used as negative controls. The number of viable, culturable GBS present in cultures over the time course was determined at selected intervals by colony counts on TSA with 5% horse blood. All growth experiments were performed in duplicate and repeated in independent experiments. Data are shown as c.f.u. ml^−1^ from one experiment, representative of several.

### Urine growth assays with iron chelation

To analyse the growth of GBS in human urine in conditions of iron chelation, growth assays were performed as described above except that urine was supplemented with 200 µm 2,2′-dipyridyl (Sigma–Aldrich, Australia) as a chelator to generate iron-limiting conditions [12,23, 24]. The 2,2′-dipyridyl was added in a final concentration of 0.2% ethanol as a carrier, which was also used in the control condition. The dependency of GBS on iron for growth was assessed by comparing strain 834, an ABU strain that grows well in urine [22], in normal and iron-limiting conditions. *E. coli* strain 83972, which utilizes iron to support growth in urine [12], was used as a control strain.

### PCR screening for genes that support GBS growth

GBS utilizes several transport systems and metabolic pathways to support growth in different conditions [25,26], and we screened for the presence of these genes in GBS 834 by PCR. The reaction conditions were optimized with 4 mM MgCl_2_ using genomic DNA from GBS reference strains A909 and COH1 that carry these genes. Primer sequences are listed in Table 1. PCR cycling conditions were as follows: initial denaturation (95 °C for 2 min), denaturation/annealing/extension (94 °C for 50 s/55 °C for 50 s/72 °C for 1 min; 35 cycles), final extension (72 °C for 4 min) and hold (4 °C).

**Table 1.** PCR primers used for screening of genes for growth in GBS 834

Primer name	Sequence	Amplicon size
*fhuD-F*	TGTCCTCACACTACTGACCTTC	605 bp
*fhuD-R*	TTAGTTCTCCACCGCGTC	
*cydA-F*	TGCACCATGATGTCCATGTTCATC	557 bp
*cydA-R*	GGGCAAATGAAGCAGAACACAAAC	
*dppA-F*	GCATTAAAGACCTCTCCATCACCT	738 bp
*dppA-R*	GGAGAGAGCGCAATAAACTTCTAG	
*dppB-F*	GAGTTACTCATGACCTTAGAGGCT	512 bp
*dppB-R*	TCCAACAGGCTTTTCCATAC	
*oppA1-F*	GGGCGTCATGTCAAAAGGA	425 bp
*oppA1-R*	CGGTAGTAGTATTATTTGTTTGATCATT	
*oppA2-F*	GGGTATATCTTTGGCGACTC	1066 bp
*oppA2-R*	CTGACCAACCTCAGCAAGAC	
*oppB-F*	CCCTATCGTTCTTTCTGATGCAGATCC	650 bp
*oppB-R*	CGCCAAAGGGCCGATTAGAGTTAA	
*oppC-F*	AGGTGCAGGATCTTCTAGCA	628 bp
*oppC-R*	GACGAGACATAGAAGTCCATCC	
*oppD-F*	TGTTGACTTCCACACATATGCTGG	723 bp
*oppD-R*	CCCCAAGTATATGGATGTTGTGGA	
*oppF-F*	GGGAGGAAAGCCTAATGAC	764 bp
*oppF-R*	CGTATAGGGGTGGATTGG	
*braB*-F	GGTATCTACTCTGGTTCTAATTGGGCG	1089 bp
*braB*-R	GGCGGCAATAGAAGTTTGGTAGACA	

### Bioinformatics

The prevalence and sequence conservation of genes involved in transport and metabolism were examined using the FASTA36 software package [27] to probe 132 complete GBS genomes on the National Center for Biotechnology Information (NCBI) database. The prevalence of genes was determined using a cut-off of >90% identity over a 95% nucleotide sequence alignment with the respective gene variants from GBS reference strain 2603 V/R [28].

### Statistical analysis

Growth curves were analysed using area-under-the-curve analysis followed by Student’s *t*-tests to compare test and control conditions. The correlation between the growth measures of OD_600 nm_ and colony counts was analysed using the Pearson correlation coefficient (*R*^2^; 0=no correlation, 1=total positive correlation). Differences in proportions of GBS isolates that exhibited robust growth versus no growth between clinical origin groups (i.e. high-grade ABU, low-grade ABU and acute UTI) were tested by chi-squared analysis. All statistical analyses were carried out using SPSS software (v26.0) and GraphPad Prism software v10.0 with significance accepted at *P*<0.05.

## Results

**Fig. 1.**
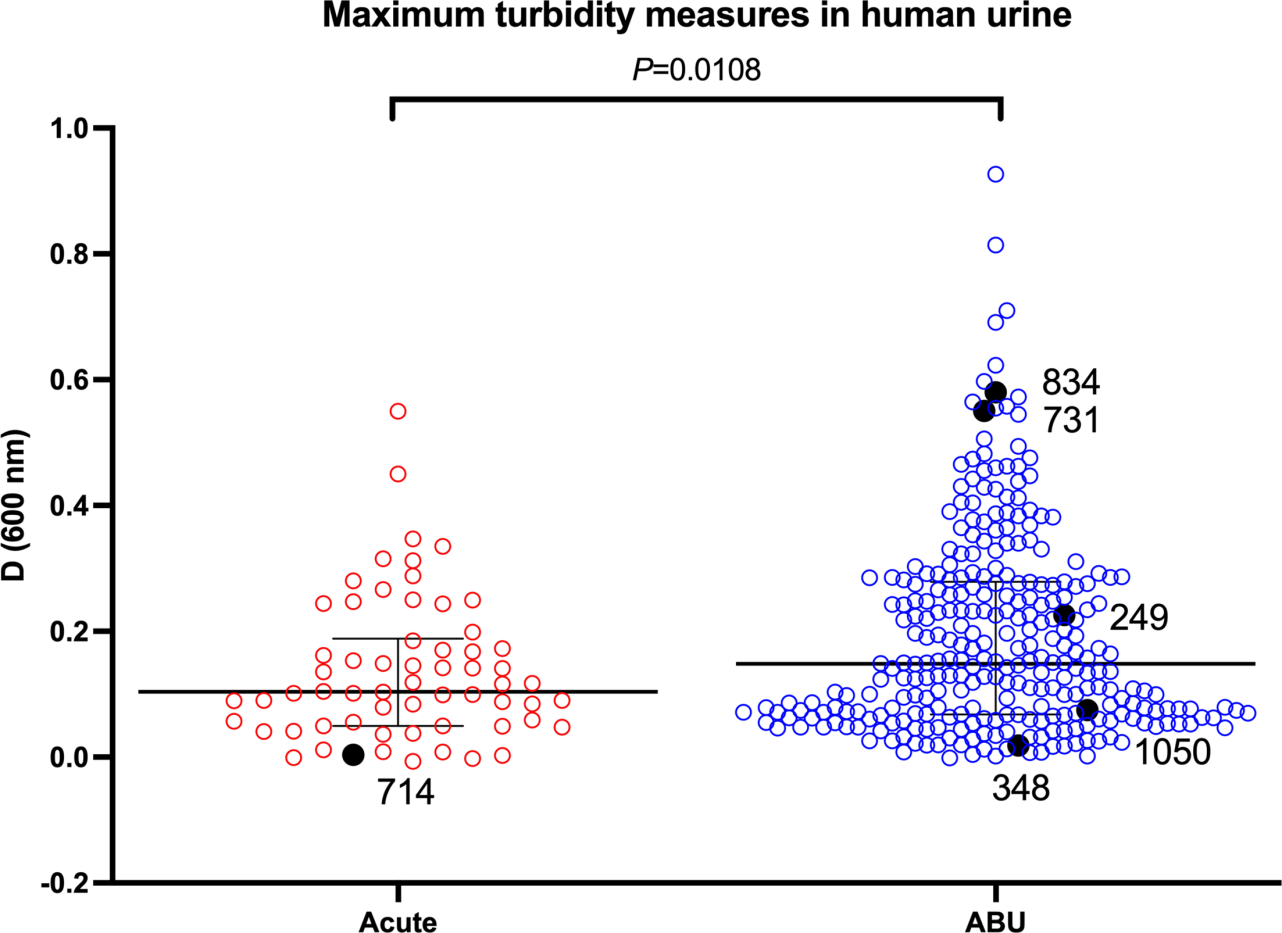
Maximum turbidity measures of cultures of acute UTI and ABU GBS strains during growth in human urine. The black dots represent the isolates shown in Fig. 2. The bars represent the median with interquartile range. Experiments were performed in duplicate, with the graphs representing one experiment.

**Fig. 2.**
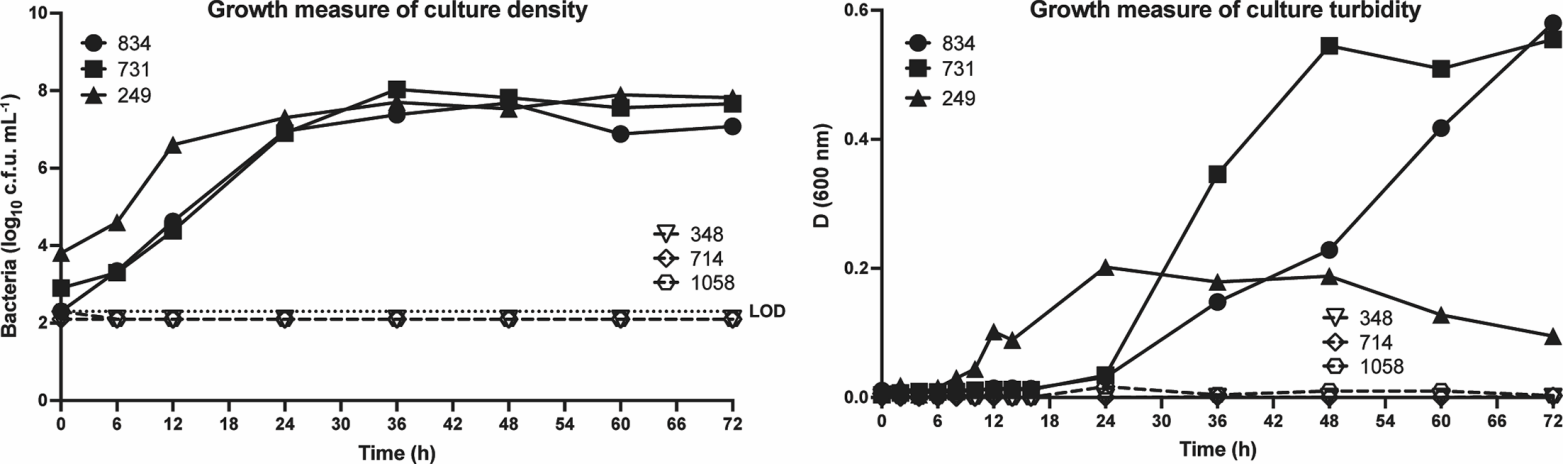
Growth of GBS in human urine. GBS isolates exhibiting robust growth in human urine (834, 731 and 249) versus no growth (348, 714 and 1058) based on (**a**) colony counts and (**b**) turbidity measurements at OD_600 nm_. The limit of detection (LOD) for colony counts was 200 c.f.u. Experiments were performed in duplicate with graphs representative of one experiment.

The 358 GBS isolates tested for growth in human urine showed a spectrum of growth phenotypes; among all isolates, 11.2% (*n*=40) exhibited robust growth in urine (defined as reaching culture density (according to viable cell counts) of >10^7^ c.f.u. ml^−1^ together with a peak culture turbidity of >0.2 OD_600 nm_ over the course of the assay). In contrast, 7.0% (*n*=25) of isolates were unable to grow in human urine (as defined by culture density <10^3^ c.f.u. ml^−1^ and <0.02 OD_600 nm_ over the course of the assay) even when analysed over an extended period of 72 h incubation. Summary data of the maximum OD_600 nm_ reached during the assay for each isolate showed significantly higher peak culture absorbance values for ABU isolates overall, compared with acute UTI isolates (*P*=0.0108; Fig. 1 and Table S1, available in the online Supplementary Material). Among the isolates that exhibited robust growth in urine and reached peak culture turbidities of >0.2 OD_600 nm_ during the assay were GBS 834, 731 and 249; among the isolates unable to grow were GBS 348, 714 and 1058; detailed growth data for these strains are illustrated in Fig. 2. Measuring the number of viable cells during growth in urine by c.f.u. estimates showed the average generation times for isolates that exhibited robust growth were similar comparing urine and THB when calculated between 0 and 36 h (average time in urine 163 vs. 145 min in THB). Taken together, these data reveal a high degree of phenotypic diversity in growth fitness for human urine of GBS when assessed across a broad, sizeable population of clinical urinary isolates.

**Fig. 3.**
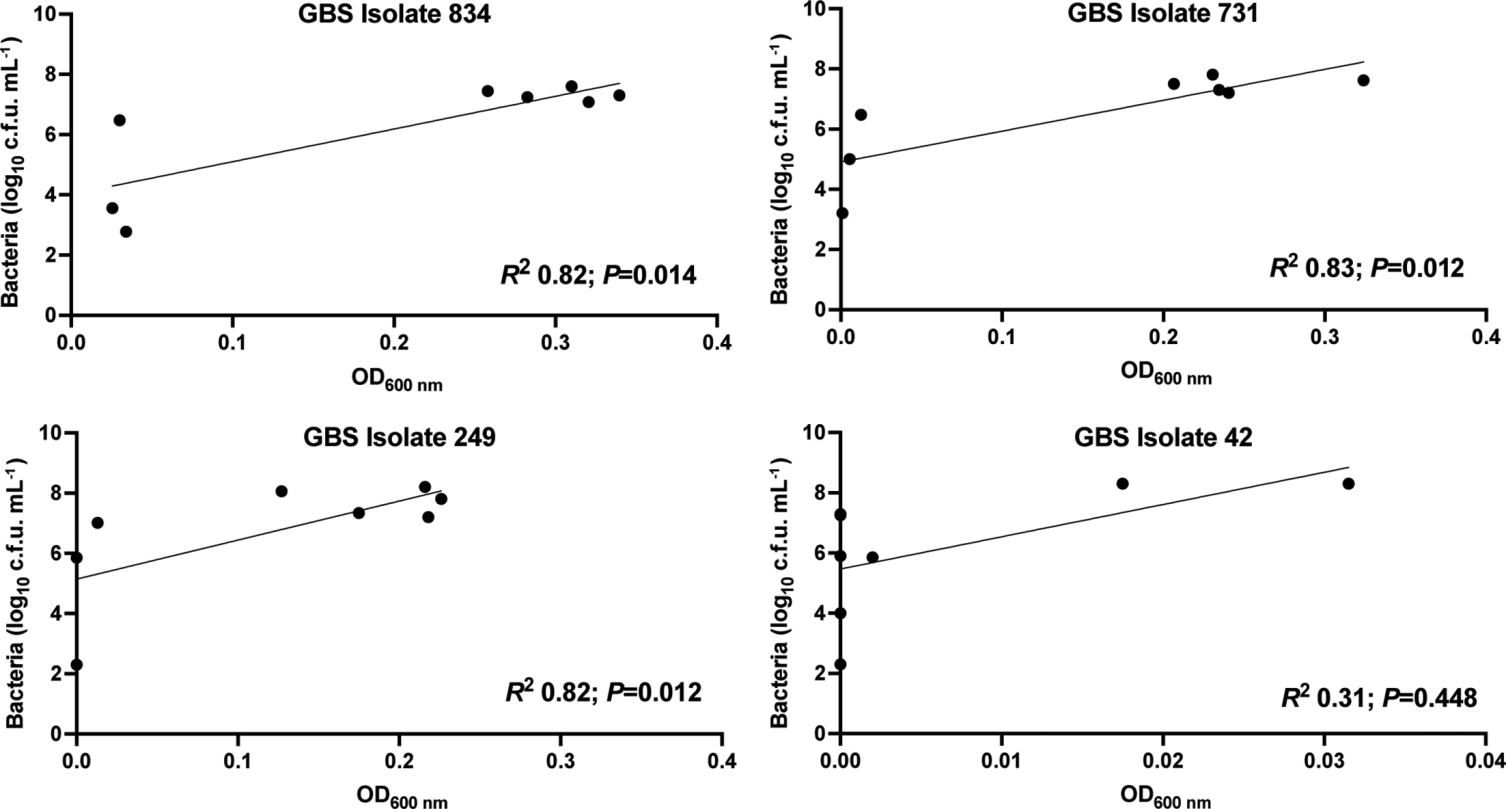
Correlation of colony count and absorbance measurements. Linear regression analyses performed using colony count estimates and OD_600 nm_ during growth in human urine show a high degree of correlation between these measures for isolates 834, 731 and 249, but not isolate 42. Analyses were performed using GraphPad Prism 10 with data from one experiment.

To evaluate the overall degree of correlation between the two growth measures of c.f.u. estimates and OD_600 nm_ readings, we compared these two datasets for all the isolates over 72 h and, surprisingly, found no significant correlation (average *R*^2^ 0.32, *n*=358). Notably, however, there was substantial variation between isolates in the degree of correlation between these two growth measures and many isolates showed a high degree of correlation between these measures; for example, GBS 834 exhibited robust growth according to both c.f.u. estimates and OD_600 nm_ readings with significant correlation (*R*^2^ 0.82, *P*=0.014; Fig. 3); similarly, we noted positive correlations (*R*^2^ >0.7) for 120 other isolates, including GBS 731 and 249 (*R*^2^ 0.825–0.831, *P*=0.012; Fig. 2). In contrast, there were low degrees of correlation between c.f.u. estimates and OD_600 nm_ readings (*R*^2^ <0.4) for 132 isolates, including GBS 42 (*R*^2^ 0.31, *P*=0.448; Fig. 3). The growth data used for correlation analysis and scatterplots (Fig. 3) are provided in Fig. S1. Taken together, these data show that concurrent measures of viable culturable GBS by colony counts and OD_600 nm_ are important to assess the phenotypic diversity of isolates for growth in urine as these measures do not consistently correlate for all isolates.

Further population analysis of all the isolates that exhibited robust growth versus no growth (and inability to survive) in urine according to the clinical origin of the isolates revealed a major difference in the likelihood of isolates being able to grow in urine based on clinical origin (acute UTI vs. ABU). A total of 40 isolates exhibited robust growth in urine in contrast to 25 isolates that showed no growth and were non-culturable after 24 h incubation; the remainder of the isolates exhibited intermediate phenotypes between these two extremes. Disproportionate representation of these 65 isolates reflected those that exhibited robust growth (*n*=40) as more likely to be associated with high-grade ABU (*n*=38) versus low-grade ABU or acute UTI (combined *n*=2) and isolates unable to grow (*n*=25) reflecting fewer (*n*=11) high-grade ABU isolates (38/40 vs. 11/25; odds ratio 4.6, 95% CI, 1.5–14.8). Of the 62 acute UTI isolates (mean bacteriuria 74 226±4068 c.f.u. ml^−1^), none grew in human urine; in contrast, 89/206 (43.2%) GBS isolates associated with high-grade ABU (mean bacteriuria 57 587±2285 c.f.u. ml^−1^) grew to high culture densities in urine. Taken together, these data show that GBS that are adapted for efficient growth in urine are almost five times more likely to cause ABU than acute UTI. Contrastingly, GBS that causes acute UTI is unable to grow (nor, in many cases, even survives) in human urine.

**Fig. 4.**
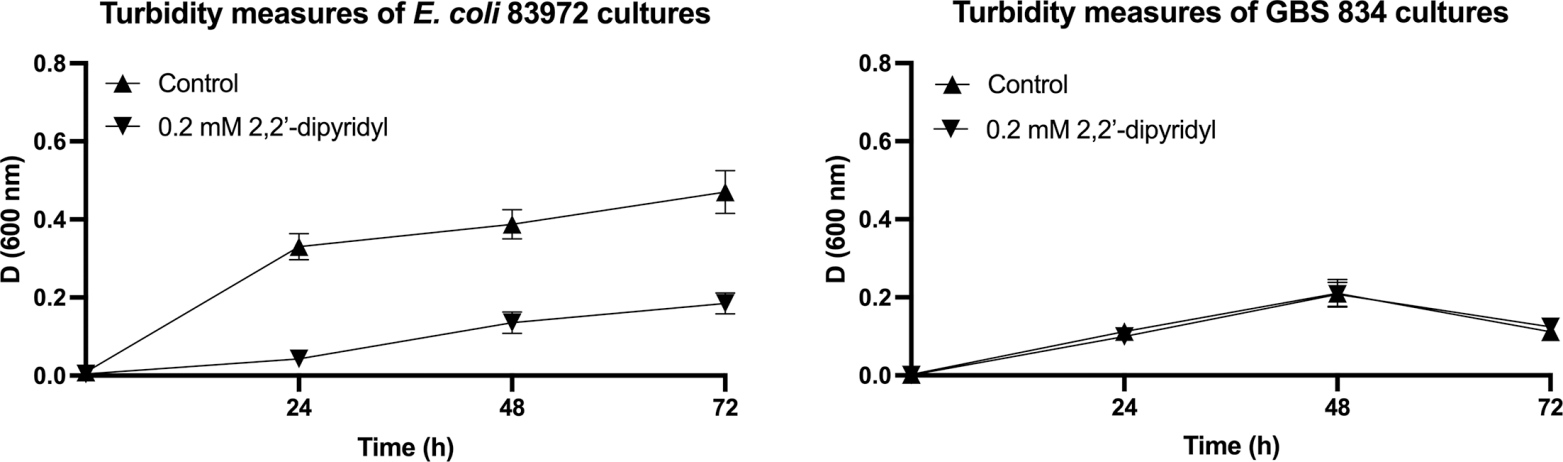
Growth of *E. coli* 83972 and GBS 834 in human urine in conditions of iron limitation. Human urine was supplemented with 2,2′-dipyridyl as an iron chelator or 0.2% EtOH in water as carrier control. The growth of *E. coli* 83972 was significantly attenuated by 0.2 mM 2,2′-dipyridyl for iron chelation (*P*<0.0001) (**a**), whereas GBS 834 exhibited no significant difference in growth between the control and iron-depleted conditions (**b**). Experiments were performed 12 independent times, with graphs showing means and sem.

To investigate whether GBS might utilize iron for growth in human urine, we performed growth experiments with GBS 834 and * E. coli* 83972 as a control strain in human urine supplemented with 2,2′-dipyridyl as an iron chelator. These assays demonstrated that in contrast to *E. coli* 83972 for which iron chelation significantly attenuated bacterial growth in urine (Fig. 4a), the growth of GBS 834 was not significantly different between control and iron-depleted conditions (Fig. 4b). To further explore this, we performed a PCR screen to examine the prevalence of several growth-related genes that could contribute to the survival and growth of GBS 834 in urine; these included genes that mediate iron transport (*fhuD*), respiration (*cydA*) and amino acid/oligopeptide transport (*dppA*, *dppB*, *oppA1-F* and *braB*). PCR confirmed the presence of all 11 genes (including *fhuD*) in GBS 834, indicating that the absence of an attenuation effect in urine in conditions of iron sequestration is not due to the lack of *fhuD*. Taken together, these findings suggest that iron utilization is not crucial for the growth of GBS 834 in human urine.

## Discussion

As an opportunistic pathogen, GBS can efficiently and asymptomatically colonize adults as a commensal in the female urogenital tract and the gastrointestinal tract [10]. In the urinary tract, GBS causes not only acute UTI, including cystitis, pyelonephritis, urethritis and urosepsis [2,7, 8], but also ABU [11]. The main finding of this study is that in a large and diverse collection of urinary GBS isolates from individuals with acute UTI and ABU, isolates that are adapted for efficient growth in human urine are much more likely to be associated with high-grade ABU than low-grade ABU or acute UTI; moreover, for ABU GBS 834, iron limitation does not drastically reduce growth in urine.

Superior fitness to utilize human urine for growth among GBS that are associated with ABU has implications for understanding the dynamics of these infections, and possibly how these bacteria may persist in the urogenital tract. For example, long-term bacteriuria appears to select for attenuated virulence phenotypes of some colonizing bacterial strains [29], and while most microbes are killed by urine, microbial growth in urine offers an opportunity for bacteria to survive and multiply in the bladder to cause persistent ABU [17]. Bacteria use several mechanisms to support survival and growth in urine, including resistance to d-serine, osmoadaptation, expression of iron acquisition systems, *de novo* synthesis of guanine and catabolism of malic acid; the latter two of which are exploited by GBS [22,30]. Future assays focusing on the factors present in human urine that ABU GBS utilize for growth will help to further define the strategies GBS uses in the urinary tract and how this relates to the conditions of ABU and UTI.

Uropathogenic bacteria express multiple virulence genes in urine [31], including genes for iron transport [32]. In *E. coli*, genes for iron utilization support growth in urine [12]. In streptococci, several species use iron acquisition to support survival and growth in nutrient-limiting environments. For example, Group A *Streptococcus* expresses proteins for haemoprotein binding and transport [33], *Streptococcus mutans* expresses a ferrous iron transport system [34] and *Streptococcus pneumoniae* has multiple iron transport systems, which are essential for virulence [20]. We investigated a potential role for the acquisition of iron by GBS in human urine to support growth by testing GBS 834 in urine supplemented with 2,2′-dipyridyl as a chelator to create iron-limiting conditions [12,23, 24]. While iron chelation significantly attenuated the growth of *E. coli* 83972 in urine over the course of 60 h, the minor difference observed in growth between chelator treatment and control for GBS 834 cultures was not statistically significant.

We then used PCR to screen for the presence of growth-related genes that might help GBS 834 survive and grow in urine: genes that play roles in iron transport (*fhuD*) [35], respiration (*cydA*) [36] and amino acid/oligopeptide transport (*dppA*, *dppB*, *oppABCDF* and *braB*) [37,38]. This analysis showed that GBS 834 harbours all the genes screened, including for iron acquisition (*fhuD*). Bioinformatic analysis of sequenced GBS isolates (*n*=132; complete GBS genomes) available on NCBI showed that all the isolates except four carry all the genes screened for in this study (Table S2). It would be interesting to analyse the expression of these genes during the growth of GBS in urine to understand how exposure to urine might alter the expression of certain metabolic pathways to support growth in this niche. Future work could also test other GBS isolates for growth in urine in conditions of iron limitation and the roles of other genes for growth in urine. Nonetheless, the results of this study suggest that iron utilization is not critical for GBS, at least for GBS strain 834, to grow in urine. Interestingly, our finding of growth of GBS 834 in urine being independent of iron is consistent with a study of GBS reference strain A909 in which GBS growth was tested in iron-limited media (THB treated with divalent cation chelator, nitrilotriacetic acid) and found to be independent of iron [35].

A strength of this study was the use of human urine to measure the growth of GBS isolates. However, human urine is highly variable in the chemical constituency and different levels of components such as glucose may influence microbial growth; for example, glucosuria enhances the growth of *E. coli*. Some bacteria such as *Enterococcus faecalis* upregulate genes for utilization of sucrose (and perhaps fructose), another constituent that is present in some urine [39,40]. The problem of variability in the constituency of human urine was partly addressed by our study design of pooling urine from six adults for each experiment and using only fresh-filtered pooled urine. Nonetheless, this study limitation is likely to have contributed to notable variability observed between assays for some isolates. An alternative to human urine is the use of synthetic human urine [41], which we excluded from this study to model the clinical condition of bacteriuria in the urinary bladder as closely as possible. Another strength of this study was the use of a large collection of over 350 clinical isolates of GBS, which provided broad insight into phenotypic traits of isolates at a population level and enabled correlations to be tested and compared across many isolates with confidence.

In summary, this study has shown that growth phenotypes of urinary GBS in human urine are disproportionately represented among isolates, whereby robust growth is more likely to be associated with high-grade ABU than low-grade ABU or acute UTI. We conclude that the ability of GBS to survive and grow in human urine is associated with the clinical condition of ABU, and for at least GBS 834, it is not significantly affected by iron bioavailability.

## supplementary material

10.1099/mic.0.001533 Uncited Table S1.

10.1099/mic.0.001533 Uncited Table S2.

10.1099/mic.0.001533 Uncited Fig. S1.

## Abbreviations

ABU: asymptomatic bacteriuria
GBS: Group B *Streptococcus*
NCBI: National Center for Biotechnology Information
TSA: tryptone soya agar
UTI: urinary tract infection

## References

[1] Paul P, Gonçalves BP, Le Doare K, Lawn JE (2023). 20 million pregnant women with group B *Streptococcus* carriage: consequences, challenges, and opportunities for prevention. Curr Opin Pediatr.

[2] Edwards MS, Baker CJ (2005). Group B streptococcal infections in elderly adults. Clin Infect Dis.

[3] Farley MM (2001). Group B streptococcal disease in nonpregnant adults. Clin Infect Dis.

[4] Goh KGK, Desai D, Thapa R, Prince D, Acharya D (2024). An opportunistic pathogen under stress: how Group B *Streptococcus* responds to cytotoxic reactive species and conditions of metal ion imbalance to survive. FEMS Microbiol Rev.

[5] Edwards MS, Rench MA, Palazzi DL, Baker CJ (2005). Group B streptococcal colonization and serotype-specific immunity in healthy elderly persons. Clin Infect Dis.

[6] Jackson LA, Hilsdon R, Farley MM, Harrison LH, Reingold AL (1995). Risk factors for group B streptococcal disease in adults. Ann Intern Med.

[7] McKenna DS, Matson S, Northern I (2003). Maternal group B streptococcal (GBS) genital tract colonization at term in women who have asymptomatic GBS bacteriuria. Infect Dis Obstet Gynecol.

[8] Ulett KB, Benjamin WH, Zhuo F, Xiao M, Kong F (2009). Diversity of group B streptococcus serotypes causing urinary tract infection in adults. J Clin Microbiol.

[9] Trivalle C, Martin E, Martel P, Jacque B, Menard JF (1998). Group B streptococcal bacteraemia in the elderly. J Med Microbiol.

[10] Shabayek S, Spellerberg B (2018). Group B streptococcal colonization, molecular characteristics, and epidemiology. Front Microbiol.

[11] Tan CK, Ulett KB, Steele M, Benjamin WH, Ulett GC (2012). Prognostic value of semi-quantitative bacteruria counts in the diagnosis of group B streptococcus urinary tract infection: a 4-year retrospective study in adult patients. BMC Infect Dis.

[12] Watts RE, Totsika M, Challinor VL, Mabbett AN, Ulett GC (2012). Contribution of siderophore systems to growth and urinary tract colonization of asymptomatic bacteriuria *Escherichia coli*. Infect Immun.

[13] Chakupurakal R, Ahmed M, Sobithadevi DN, Chinnappan S, Reynolds T (2010). Urinary tract pathogens and resistance pattern. J Clin Pathol.

[14] Gordon DM, Riley MA (1992). A theoretical and experimental analysis of bacterial growth in the bladder. Mol Microbiol.

[15] Roos V, Ulett GC, Schembri MA, Klemm P (2006). The asymptomatic bacteriuria *Escherichia coli* strain 83972 outcompetes uropathogenic E. coli strains in human urine. Infect Immun.

[16] Stamey TA, Mihara G (1980). Observations on the growth of urethral and vaginal bacteria in sterile urine. J Urol.

[17] Ipe DS, Horton E, Ulett GC (2016). The basics of bacteriuria: strategies of microbes for persistence in urine. Front Cell Infect Microbiol.

[18] Ipe DS, Sundac L, Benjamin WH, Moore KH, Ulett GC (2013). Asymptomatic bacteriuria: prevalence rates of causal microorganisms, etiology of infection in different patient populations, and recent advances in molecular detection. FEMS Microbiol Lett.

[19] Skaar EP, Humayun M, Bae T, DeBord KL, Schneewind O (2004). Iron-source preference of *Staphylococcus aureus* infections. Science.

[20] Brown JS, Holden DW (2002). Iron acquisition by Gram-positive bacterial pathogens. Microbes Infect.

[21] Mabbett AN, Ulett GC, Watts RE, Tree JJ, Totsika M (2009). Virulence properties of asymptomatic bacteriuria *Escherichia coli*. Int J Med Microbiol.

[22] Ipe DS, Ben Zakour NL, Sullivan MJ, Beatson SA, Ulett KB (2016). Discovery and characterization of human-urine utilization by asymptomatic-bacteriuria-causing *Streptococcus agalactiae*. Infect Immun.

[23] Nakouti I, Hobbs G (2013). A new approach to studying ion uptake by actinomycetes. J Basic Microbiol.

[24] Nakouti I, Sihanonth P, Hobbs G (2012). A new approach to isolating siderophore-producing actinobacteria. Lett Appl Microbiol.

[25] Sitkiewicz I, Musser JM (2009). Analysis of growth-phase regulated genes in *Streptococcus agalactiae* by global transcript profiling. BMC Microbiol.

[26] Mereghetti L, Sitkiewicz I, Green NM, Musser JM (2008). Extensive adaptive changes occur in the transcriptome of *Streptococcus agalactiae* (group B *Streptococcus*) in response to incubation with human blood. PLoS One.

[27] Pearson WR, Lipman DJ (1988). Improved tools for biological sequence comparison. Proc Natl Acad Sci USA.

[28] Tettelin H, Masignani V, Cieslewicz MJ, Eisen JA, Peterson S (2002). Complete genome sequence and comparative genomic analysis of an emerging human pathogen, serotype V *Streptococcus agalactiae*. Proc Natl Acad Sci USA.

[29] Salvador E, Wagenlehner F, Köhler C-D, Mellmann A, Hacker J (2012). Comparison of asymptomatic bacteriuria *Escherichia coli* isolates from healthy individuals versus those from hospital patients shows that long-term bladder colonization selects for attenuated virulence phenotypes. Infect Immun.

[30] Ipe DS, Sullivan MJ, Goh KGK, Hashimi SM, Munn AL (2021). Conserved bacterial de novo guanine biosynthesis pathway enables microbial survival and colonization in the environmental niche of the urinary tract. ISME J.

[31] Shepard BD, Gilmore MS (2002). Differential expression of virulence-related genes in *Enterococcus faecalis* in response to biological cues in serum and urine. Infect Immun.

[32] Vebo HC, Solheim M, Snipen L, Nes IF, Brede DA (2010). Comparative genomic analysis of pathogenic and probiotic *Enterococcus faecalis* isolates, and their transcriptional responses to growth in human urine. PLoS One.

[33] Bates CS, Montañez GE, Woods CR, Vincent RM, Eichenbaum Z (2003). Identification and characterization of a *Streptococcus pyogenes* operon involved in binding of hemoproteins and acquisition of iron. Infect Immun.

[34] Evans SL, Arceneaux JE, Byers BR, Martin ME, Aranha H (1986). Ferrous iron transport in *Streptococcus mutans*. J Bacteriol.

[35] Clancy A, Loar JW, Speziali CD, Oberg M, Heinrichs DE (2006). Evidence for siderophore‐dependent iron acquisition in group B streptococcus. Mol Microbiol.

[36] Yamamoto Y, Poyart C, Trieu-Cuot P, Lamberet G, Gruss A (2005). Respiration metabolism of Group B *Streptococcus* is activated by environmental haem and quinone and contributes to virulence. Mol Microbiol.

[37] Samen U, Gottschalk B, Eikmanns BJ, Reinscheid DJ (2004). Relevance of peptide uptake systems to the physiology and virulence of *Streptococcus agalactiae*. J Bacteriol.

[38] Sitkiewicz I, Green NM, Guo N, Bongiovanni AM, Witkin SS (2009). Transcriptome adaptation of group B *Streptococcus* to growth in human amniotic fluid. PLoS One.

[39] Tasevska N, Runswick SA, McTaggart A, Bingham SA (2005). Urinary sucrose and fructose as biomarkers for sugar consumption. Cancer Epidemiol Biomarkers Prev.

[40] Bouatra S, Aziat F, Mandal R, Guo AC, Wilson MR (2013). The human urine metabolome. PLoS One.

[41] Ipe DS, Ulett GC (2016). Evaluation of the in *vitro* growth of urinary tract infection-causing Gram-negative and Gram-positive bacteria in a proposed synthetic human urine (SHU) medium. J Microbiol Methods.

