# Supplementary material for: Group B Streptococcus growth in human urine is associated with asymptomatic bacteriuria rather than urinary tract infection and is unaffected by iron sequestration
Source: Microbiology (Reading). 2025 Feb 20;171(2):001533. doi: 10.1099/mic.0.001533 (PMC11842879; doi:10.1099/mic.0.001533)
Supplement: Uncited Fig. S1. [file mic-171-01533-s003.pdf]

Group B Streptococcus growth in human urine is associated with asymptomatic bacteriuria rather than urinary tract infection and unaffected by iron sequestration

Deepak S Ipe[1*], Kelvin G K Goh[1*], Devika Desai[1], Nouri Ben-Zakour[2], Matthew J Sullivan[1,†], Scott A Beatson[2] and Glen C Ulett[1‡]

Supplementary Material.

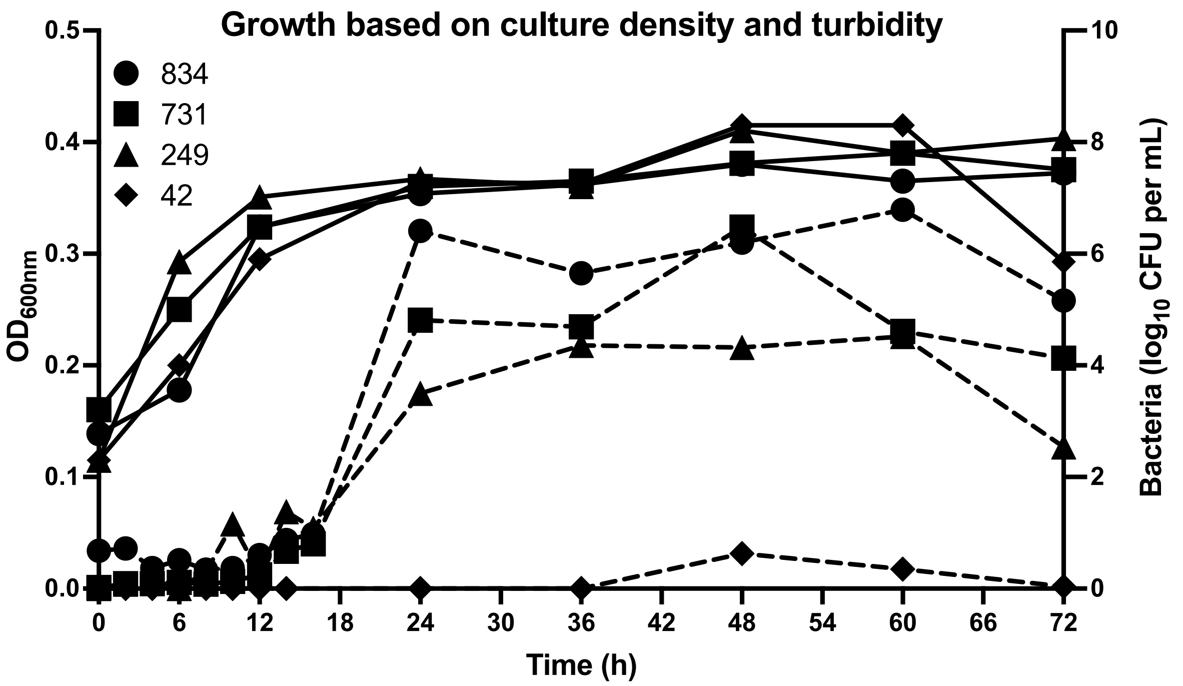

Supplementary Figure 1. Growth of GBS in human urine. GBS growth based on turbidity measurements at OD_{600nm} (solid lines) or colony counts (dashed lines) used for linear regression analyses. Experiments were performed in duplicate, with graphs representative of one experiment.
